# Transcription Analysis for Core Networks of lncRNAs–mRNAs: Implication for Potential Role in Sterility of *Crassostrea gigas*

**DOI:** 10.3390/biology11030378

**Published:** 2022-02-27

**Authors:** Huihui Wang, Hong Yu, Qi Li, Shikai Liu

**Affiliations:** 1 Key Laboratory of Mariculture (Ministry of Education), Ocean University of China, Qingdao 266003, China; 21200511137@stu.ouc.edu.cn (H.W.); qili66@ouc.edu.cn (Q.L.); liushk@ouc.edu.cn (S.L.); 2 Laboratory for Marine Fisheries Science and Food Production Processes, Pilot National Laboratory for Marine Science and Technology (Qingdao), Qingdao 266237, China; 3 Laboratory of Tropical Marine Germplasm Resources and Breeding Engineering, Sanya Oceanographic Institution, Ocean University of China, Sanya 572000, China

**Keywords:** core lncRNA–mRNA networks, *Crassostrea gigas*, triploid sterility

## Abstract

**Simple Summary:**

This study reveals the expression profiles of lncRNA in the gonads of the Pacific oyster *Crassostrea gigas*. The potential function of lncRNAs was predicted in the case of antisense and cis-regulatory mechanisms based on their physical positions and their coexpression relationships in the case of trans regulation. Sterility-related DEGs and DELs were chosen for subsequent analysis, demonstrating that trans-regulatory lncRNAs might play a vital role in the gametogenesis of *C. gigas*. We constructed core networks of lncRNAs–mRNAs for triploid sterile females and hermaphrodites based on pathway results, in which 28 lncRNAs and their 54 trans-regulatory genes were detected. Among 28 sterility-specific lncRNAs, MSTRG.79882.3 and MSTRG.79882.4 for triploid sterile females and MSTRG.33704.1, MSTRG.63844.1, and MSTRG.5675.1 for hermaphrodites play the most significant role.

**Abstract:**

Long noncoding RNA (lncRNA), a type of non-protein-coding transcript, is emerging as a crucial regulator of gene expression. However, few roles of lncRNA in the reproductive process of the Pacific oyster (*Crassostrea gigas*) have been defined, especially in the regulatory mechanism of sterile triploids gametogenesis. To uncover the potential role of lncRNA, the gonads of diploids, sterile triploids, and partially sterile triploids underwent RNA sequencing. A total of 9618 reliable lncRNAs were identified. The target relationship between lncRNA and mRNA was predicted based on cis, trans, and antisense regulation with bioinformatic software. We chose differentially expressed lncRNAs and mRNAs when sterile triploids were compared to partially sterile triploids and diploids for subsequent functional enrichment analysis. Findings revealed that trans-regulatory lncRNAs might play a significant role in the gametogenesis of *C. gigas*. Combining pathway results, we constructed core networks of lncRNAs–mRNAs for triploid sterile females and hermaphrodites. Fifty-four genes related to cell division, germline-cell maintenance, and glycogen metabolism were found to be associated with sterility. A total of 28 candidate lncRNAs were predicted to trans-regulate these genes. We speculated that MSTRG.79882.3 and MSTRG.79882.4 for triploid sterile females and MSTRG.33704.1, MSTRG.63844.1, and MSTRG.5675.1 for hermaphrodites were highly important as they were predicted to regulate more sterility-specific genes than others. Our work collectively identified sterility-related lncRNAs and implicated the potential mechanism of lncRNA-mediated regulation in the gametogenesis of sterile triploid oysters.

## 1. Introduction

Gametogenesis is an integral part of the reproductive process, in which germ cells undergo proliferation and differentiation, thereby releasing their genetic information to the next generation. Compared to diploids, triploids need to overcome meiosis abnormalities during gametogenesis because of a redundant set of chromosomes [1]. Most aquaculture triploids of great commercial value have been reported to be sterile, and thus, triploids could represent an interesting model for studies on gametogenesis.

lncRNAs are RNA transcripts longer than 200 nucleotides that are not translated into polypeptides. Based on their loci and relationship with the functional genes on the chromosome, lncRNAs are classified as intronic, intergenic, sense, bidirectional, and antisense [2]. Functionally, lncRNAs were characterized into cis long noncoding RNAs (cis-lncRNAs) that regulate local gene expression and trans long noncoding RNAs (trans-lncRNAs) that function as cellular factor leaves the site of itself [3]. Antisense long noncoding RNAs (antisense-lncRNAs), as a sub-class of lncRNAs transcribed from the antisense of a protein-coding strand, have also been broadly investigated [4].

The function of the majority of lncRNAs remains enigmatic; nonetheless, emerging evidence has shown that lncRNAs take effect in various reproduction biological processes, including sex determination [5], reproduction [6], and ovulation [7]. To date, a large number of studies on lncRNAs functionally involved in gametogenesis have been performed. The successful application of the CRISPR-mediated knockout technology in model organisms has directly revealed the mechanism of specific lncRNAs functioning in the gamete maturation process [8]. For species with incomplete annotation of noncoding RNA, screening out candidate lncRNAs is therefore of great importance. Differentially expressed lncRNAs associated with oocyte meiosis, homologous recombination, and regulating triploid fertility were identified in rainbow trout through miRNA–mRNA/lncRNA network construction [9]. Despite the surging interest in the function of lncRNAs in reproduction, investigations involving its systematic identification and description in marine invertebrate gametogenesis are lacking.

The Pacific oyster *Crassostrea gigas* is a representative marine bivalve for studying gametogenesis due to the complex sterility trait of triploids, as described foremostly by Jouaux et al. [10]. The gametogenic pattern of triploids histologically can be roughly divided into α-pattern and β-pattern. Accordingly, the triploid with α-pattern was named 3nα, which tends to produce abundant gametes at the mature stage, and some with the latter pattern were named 3nβ, possessing few abnormal gametes at sexual maturity. In addition, a significant number of triploid hermaphrodites were more common than diploids. A later study of triploid *Crassostrea virginica* enriched the classification system of gonad development [11]. A recent study has refined the classification system of *C. gigas*, in which triploids were divided into female (female α and female β), male, and hermaphrodites [12]. It was demonstrated that male 3nβ is mainly represented by hermaphrodites with experimental observations.

However, histological observation alone cannot unravel the essence, and in-depth exploration of the mechanisms underlying sterility should be explored. During the annual reproductive cycle, vesicular connective tissue cells of the oyster gonad serve the function of accumulating nutrients, such as lipids and glycogen, and satisfying reproductive energy needs [13]. Allocating less energy to gametogenesis may account for partial triploid infertility but superior growth relative to diploids [14]. Yue et al. [15] reported that *CgBHMG1*, a factor involved in meiosis, was aberrantly expressed in the gonad of triploids. Further, transcriptomic and proteomic analysis results unveiled that cell-division-related genes were downregulated in sterile oysters, while the genes involved in nutrient biosynthesis and cell apoptosis were upregulated [16,17]. It confirmed that energy-metabolism difference and cell cycle arrest, to some extent, do induce sterility of *C. gigas*. Although previous studies have identified some sterility-related genes, whether the noncoding transcripts regulate genes of interest remains elusive.

Thus, there is an urgent need to pinpoint sterility-specific lncRNAs and investigate their potential functions in the gametogenesis of *C. gigas*. In this study, the gonads of diploid female (F-2n), diploid male (M-2n), triploid female with α-pattern (F-3nα), triploid female with β-pattern (F-3nβ), triploid male with α-pattern (M-3nα), and sterile hermaphrodites (H-3n) were used for RNA sequencing. Subsequently, gonad-specific lncRNAs were systematically identified, and the primary characterization, expression pattern, and regulatory mechanisms of these lncRNAs were predicted and analyzed. Further, we detected the differentially expressed genes involved in the pathways of interest and the lncRNAs that interact with them. The core networks of lncRNAs–mRNAs whereby these interactions occur were constructed. Our results provide a new perspective for studying sterility and further expand our vision for the regulatory role of lncRNAs.

## 2. Materials and Methods

### 2.1. Animals and Sample Collection 

Triploid oysters were obtained by mating diploid females and tetraploid males in a private hatchery in 2017. Ploidy of F-2n, M-2n, F-3nα, F-3nβ, M-3nα, and H-3n was detected by flow cytometry, and gonadal staging and gender determination were performed by histology. All samples were instantly frozen in liquid nitrogen and then stored at −80 °C before RNA isolation.

### 2.2. RNA Extraction, Library Preparation, and RNA Sequencing

In the present study, 18 strand-specific cDNA libraries of triploid and diploid oysters were constructed with three bio-replicates in each gonad type, followed by quality control, ribosomal alignment, reference genome alignment, and transcript reconstruction. The gonad RNA was extracted with Trizol (Invitrogen, Carlsbad, CA, USA), and then genomic DNA was dragged using DNase I (New England Biolabs). The purity of total RNA was tested by Agilent 2100 Bioanalyzer (Agilent Technologies, Palo Alto, CA, USA) and checked with RNase-free agarose gel electrophoresis. After ribosome removal, retained mRNAs and ncRNAs were broken into short fragments with fragmentation buffer. The first strand of cDNA was synthesized using the fragmented RNA as a template with random hexamers. Then, buffer solution, dNTPs (dUTP instead of dTTP), DNA polymerase I, and RNase HH were added to synthesize the second cDNA strand. The cDNA was purified by QIAquick PCR extraction kit (Qiagen, Venlo, The Netherlands), repaired at the end, base A. Next, the second chain was degraded by uracil-N-glycosylase (UNG). Segment size selection was performed by agarose gel electrophoresis for PCR amplification. Finally, 18 strand-specific cDNA libraries with three biological replicates were assembled and sequenced with Illumina HiSeq 4000 by Gene Denovo Biotechnology Co. (Guangzhou, China). All raw data were submitted to the NCBI sequence read archive (SRA) database under accession number PRJNA690125 (https://www.ncbi.nlm.nih.gov/sra/?term=PRJNA690125, accessed on 6 January 2021).

### 2.3. lncRNA Identification and Differential Expression Analysis

Quality checks of raw sequence reads were executed by fastp (version v0.18.0). (https://github.com/OpenGene/fastp, accessed on 12 December 2019). Furthermore, low-quality reads were discarded [18]. Reads were compared with HISAT2 (version v2.1.0) to the reference genome (unpublished) [19] after using the short reads comparison tool Bowtie2 (version v2.2.8) [20] to remove partial residual rRNA. Transcripts of each sample were then reconstructed by StringTie (version v1.3.4) [21,22]. Coding potential calculator (CPC, version v0.9-r2) and coding non-coding index (CNCI, version v2) (http://cpc.cbi.pku.edu.cn/, accessed on 12 December 2019) were used to predict the coding ability of the new transcripts, and the newly predicted lncRNAs were obtained [23,24]. lncRNAs were classified into five types based on their location relative to protein-coding genes: intergenic, bidirectional, intronic, antisense, and sense overlapping lncRNAs. Read count and FPKM (fragments per kilobase of transcript per million mapped reads) value were calculated using StringTie software. Differential expression analysis was performed by DESeq2 [25]. The |log2 (fold change)| ≥ 1 and FDR < 0.05 were used as the significance threshold to identify differentially expressed genes. For heatmap plots, we used Heatmap (version v1.0.12).

### 2.4. Target Gene Prediction of lncRNAs and Functional Analysis of mRNAs

The complementary binding and the optimal base-pairing relationship between antisense lncRNA and mRNA were predicted by RNAplex (version v0.2) (http://www.tbi.univie.ac.at/RNA/RNAplex.1.html, accessed on 12 December 2019) [26] and R package ViennaRNA [27]. Additionally, lncRNAs located within 10 kb upstream or downstream of mRNAs were predicted to have a cis function in regulating neighboring protein-coding genes. Trans regulation was identified by calculating the Pearson correlation coefficients based on the expression level. Finally, mRNAs with absolute correlation coefficients greater than 0.999 were selected for functional enrichment analysis to predict the functions of lncRNA. The Gene Ontology (GO) and Kyoto Encyclopedia of Genes and Genomes (KEGG) analyses were subsequently conducted. GO terms and KEGG pathways with *p*-value < 0.05 were significantly enriched. The core networks were constructed with candidate lncRNAs and mRNAs by Gene Denovo Omicshare online tools (https://www.omicshare.com/tools/, accessed on 25 November 2021).

### 2.5. qRT-PCR Validation of RNA-seq Data

Using PrimerScript RT reagent kit with gDNA Eraser (Takara, Dalian, China), RNA was reverse transcribed to form first-strand cDNA from 1 μg total RNA. The primers were designed using Primer Premier v.5.0 software (Premier Biosoft International, San Francisco, CA, USA) and are shown in Table 1. The quantitative real-time PCR was conducted using QuantiNova SYBR Green PCR Kit (Qiagen, Hilden, Germany) on the LightCycler 480 real-time PCR instrument (Roche Diagnostics, Burgess Hill, UK). The qRT-PCR reaction contained 5 μL 2× SYBR Green PCR Master Mix, 1 μL specific forward primer (10 μM), 1 μL specific reverse primer (10 μM), and 1 μL diluted cDNA template, and 3 μL RNase-free water was added to a final volume of 10 μL. All reactions were performed in triplicate for each sample with cycling parameters consisting of 95 °C for 3 min; followed by 35 cycles of 95 °C for 15 s, 60 °C for 15 s, and 72 °C for 10 s; and finally up to 95 °C for 15 s. Elongation factor I (EF I) gene expression was used to normalize the target gene expression. The relative gene expression level was calculated by the 2^−ΔΔCt^ method. Data were analyzed by *t*-test using software SPSS 18.0, and statistical significance was determined at *p*-value < 0.05.

## 3. Results

### 3.1. Upstream Preprocessing of RNA-Sequencing Data

**Figure 1:**
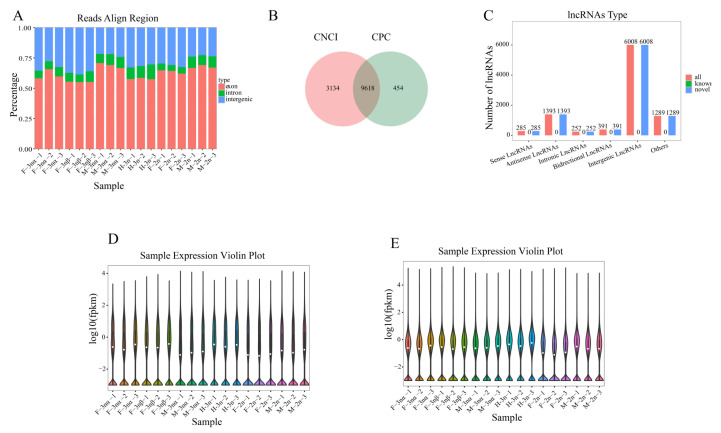
Sequencing and data preprocessing of *C. gigas* gonad. (**A**) The distribution position in the reference genome of all reads was divided into exon, intron, and intergenic regions. (**B**) The number of reliable lncRNA was predicted with CPC and CNCI software. (**C**) The number of different types of lncRNAs. (**D**,**E**) Violin diagrams showing expression levels of genes and lncRNAs in each sample.

Each sequenced sample had 7.7 to 10.2 million clean reads after data filtering. A range of 61% to 84% total reads was mapped to the reference genome, and the mapped rates were around 80% (Table S1). According to the distribution position in the reference genome, all reads were divided into exon, intron, and intergenic regions. Our results indicated that most of the reads were compared to the exon region (Figure 1A). Combining the results of CPC and CNCI, we obtained 9618 reliably expressed lncRNAs (all novel) (Figure 1B). Of these lncRNAs, 285 sense, 1393 antisense, 391 antisense, antisense lncRNAs, 252 intronic, and 6008 intergenic were identified (Figure 1C). A total of 31,172 mRNAs (29,843 known and 1329 novel) were identified after transcript reconstruction. The expression distribution of lncRNA and mRNA demonstrated that data from 18 samples could be used for further analysis (Figure 1D,E).

### 3.2. Identification of DELs and DEGs

**Figure 2:**
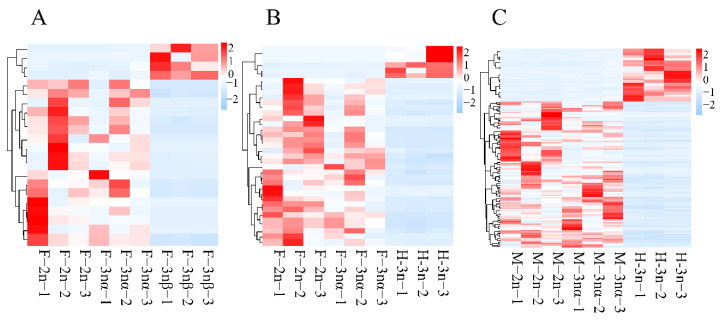
Transcript patterns of DELs in F-3nβ group (**A**), HF-3n group (**B**), and HM-3n group (**C**) are presented in heatmaps. Red indicates relatively high expression, while blue indicates relatively low expression among samples.

**Figure 3:**
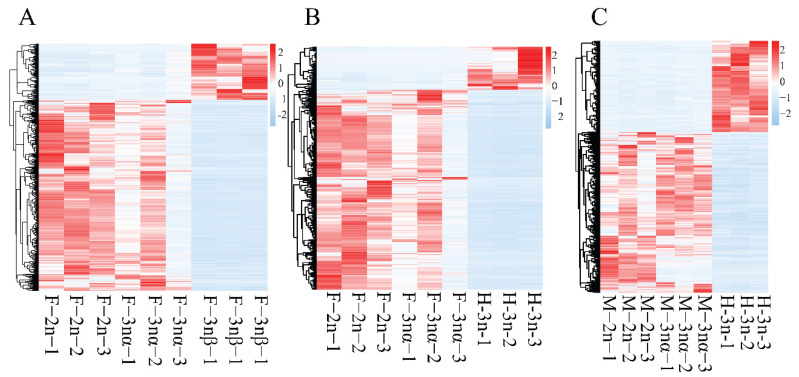
Transcript patterns of DEGs in F-3nβ group (**A**), HF-3n group (**B**), and HM-3n group (**C**) are presented in heatmaps. Red indicates relatively high expression, while blue indicates relatively low expression among samples.

Differential expression analysis resulted in the identification of 262, 43, 426, 53, 334, and 448 DELs and 5132, 1183, 4973, 866, 2288, and 4081 DEGs in F-3nβ-vs-F-2n, F-3nβ-vs-F-3nα, H-3n-vs-F-2n, H-3n-vs-F-3nα, H-3n-vs-M-2n, and H-3n-vs-M-3nα, respectively (Tables S2 and S3). To get a clearer perspective of the role played by the lncRNA–mRNA core networks in triploid sterile individuals, we initiated our investigation in the F-3nβ group (F-3nβ-vs-F-2n and F-3nβ-vs-F-3nα), HF-3n group (H-3n-vs- F-2n and H-3n-vs-F-3nα), and HM-3n group (H-3n-vs-M-2n and H-3n-vs-M-3nα) separately. Among 22 lncRNAs differentially expressed in F-3nβ as compared to F-2n and F-3nα, 4 DELs were upregulated and 18 were downregulated in F-3nβ (Figure 2A). Of the 37 DELs in the HF-3n group, 6 lncRNAs were upregulated and 31 were downregulated in H-3n compared to female fertile individuals (Figure 2B). One hundred fifty-one lncRNAs were differentially expressed in HM-3n group, with 40 upregulated and 111 downregulated in H-3n (Figure 2C). In addition, 931, 675, and 1464 DEGs were detected in F-3nβ group, HF-3n group, and HM-3n group, respectively. The expression profiles of these DEGs are shown with heatmaps (Figure 3A–C). The heatmaps highlight that both DEGs and DELs showed unified expression profiles between sterile oysters and fertile individuals.

### 3.3. Target Gene Prediction of Differentially Expressed lncRNAs

**Figure 4:**
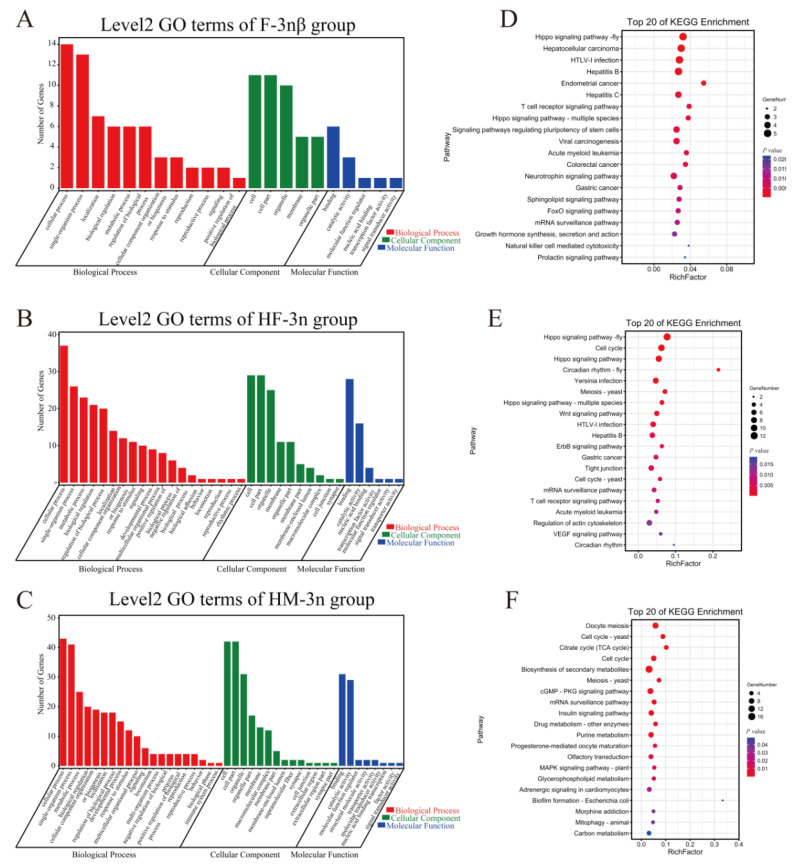
Gene Ontology (GO) enrichment analysis of differentially expressed genes of *C. gigas* revealed highly enriched biological processes associated with detected DEGs (**A**–**C**). Based on the GO results, cellular process, DNA binding, and catalytic activity were the most enriched GO terms in sterile oysters compared to fertile ones. KEGG enrichment of differentially expressed genes with top 20 terms (**D**–**F**); “GeneNumber” is the number of genes that were enriched in the term; “RichFactor” refers to the ratio of the number of genes in the term to the total number of genes.

To accurately understand the mechanism of regulation of target mRNAs by lncRNAs, we categorized lncRNA loci into those that might regulate gene expression in cis, perform functions in trans, and participate in the regulation post-transcriptional processes in an antisense role. We obtained 1561 pairs of 1393 regulatory antisense lncRNAs with 1288 matched mRNAs (Table S4), of which 2, 74, and 2 pairs of DELs–DEGs were observed in F-3nβ, HF-3n, and HM-3n groups, respectively. Additionally, 52 unique DEGs, which participated in 47 pairs of antisense regulation in the HF-3n group, were notably enriched for progesterone-mediated oocyte maturation, apoptosis, TNF signaling pathway, and cAMP signaling pathway. Subsequently, a total of 11591 pairs of cis-regulatory associations were observed (Table S5). We detected 1, 4, and 29 pairs of DEGs–DELs in the F-3nβ, HF-3n, and HM-3n groups, respectively. Interestingly, we found that TRPM3 is a cis-target gene of lncRNA MSTRG.19956.1 for both the F-3nβ group and the HF-3n group; Tex36 is cis-targeted by lncRNA MSTRG.22725.2 for both the HF-3n group and the HM-3n group. Finally, 7951 pairs of lncRNAs–mRNAs that act as trans-regulatory relationships were presented (Table S6). Notably, we detected more DEL–DEG pairs in all three groups, with 139 pairs in F-3nβ, 545 pairs in HF-3n, and 950 pairs in HM-3n. To functionally explore trans regulation in sterility, the unique DEGs, with 100 in the F-3nβ group, 267 in the HF-3n group, and 451 in the HM-3n group, were annotated with GO and KEGG terms. The GO analysis results indicated that trans-acting genes were classified into similar subcategories in three groups, such as cellular process and single-orgasm process (biological processes), cell and cell part (cellular components), and binding and catalytic activity (molecular functions) (Figure 4A–C). Meanwhile, KEGG analysis demonstrated that cell division and apoptosis might account for the sterility (Figure 4D–F). Collectively, these findings indicated that lncRNAs could potentially regulate genes via trans action to participate in the gametogenesis of *C. gigas*.

### 3.4. Exploration of the Core Networks of lncRNAs–mRNAs

**Figure 5:**
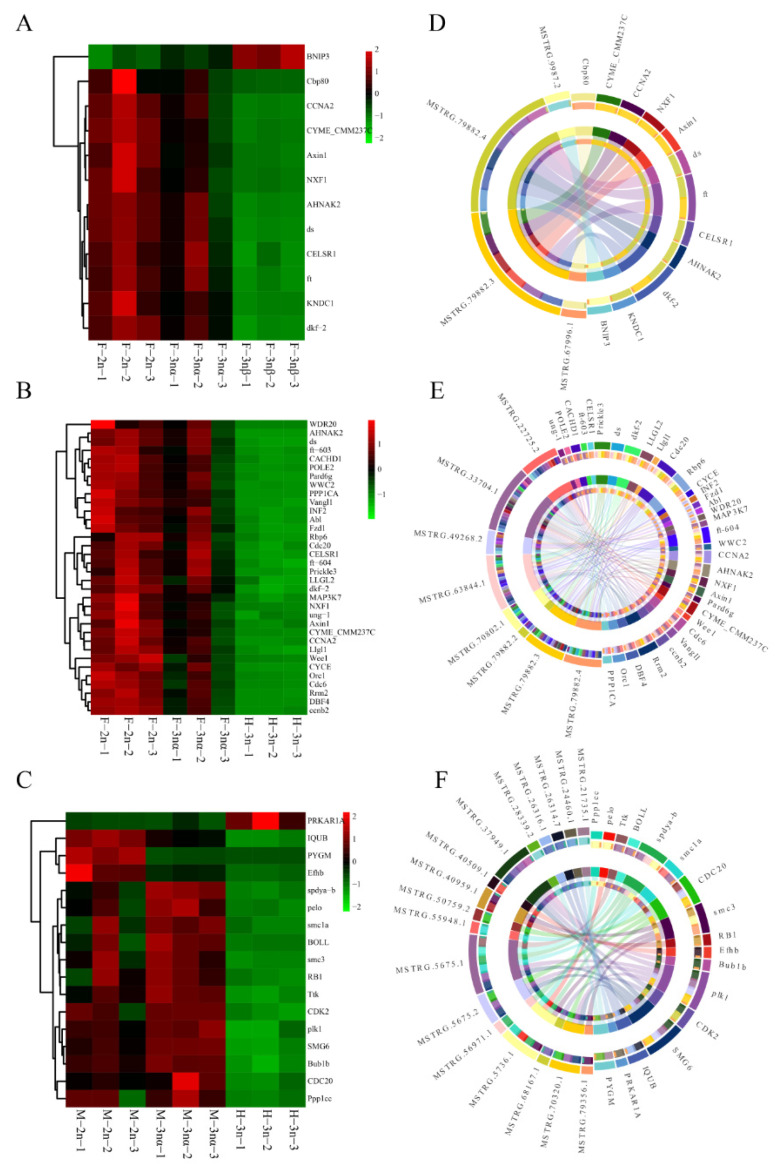
Identification of core lncRNA–mRNA networks for the gametogenesis arrest of sterile triploid oysters. The expression of core genes involved in the pathways of interest in F-3nβ group (**A**), HF-3n group (**B**), and HM-3n group (**C**); the core networks of lncRNA–mRNA of F-3nβ group (**D**), HF-3n group (**E**), and HM-3n group (**F**); targeted genes are listed in the right hemisphere, lncRNAs in the left hemisphere, and the interline represents targeted relationships.

We focused on trans regulation, combining functional pathway results to establish core networks of lncRNAs–mRNAs in three groups. In the F-3nβ group, the significantly enriched pathways (*p*-value < 0.05) of interest include Hippo signaling pathway—multiple species, progesterone-mediated oocyte maturation, FoxO signaling pathway, mRNA surveillance pathway, and Jak-STAT signaling pathway (not all of them are shown in the Figure 4D). Twelve mRNAs (with annotated gene symbols) enriched in the above pathways were targeted by upregulated lncRNA MSTRG.9987.2 and downregulated lncRNAs MSTRG.79882.4, MSTRG.79882.3, and MSTRG.67996.1. In addition, BNIP3, the target gene of MSTRG.9987.2, was upregulated while others were downregulated in F-3nβ (Figure 5A). Among these four lncRNAs, MSTRG.79882.4 and MSTRG.79882.3 were two for which it is worth noting lncRNAs with nine genes under their regulation. In the HF-3n group, there were 34 trans-regulatory genes (with annotated gene symbols), containing CCNA2, Cdc6, and ccnb2. They were significantly enriched (*p*-value < 0.05) for cell cycle, Hippo signaling pathway, Hippo signaling pathway—multiple species, wnt signaling pathway, mRNA surveillance pathway, base excision repair, p53 signaling pathway, oocyte meiosis, and progesterone-mediated oocyte maturation (not all of them are presented in the Figure 4E). The expression profiles indicated that these 34 unique genes were all downregulated in H-3n (Figure 5B). Not surprisingly, the eight lncRNAs potentially regulating these genes were also downregulated in H-3n. In the HM-3n group, pathways of oocyte meiosis, cell cycle, mRNA surveillance pathway, insulin signaling pathway, and progesterone-mediated oocyte maturation were paid attention to. We further identified 17 unique mRNAs that were functionally accumulated in the process above (*p*-value < 0.05). Among them, PRKAR1A, which was targeted by lncRNA MSTRG.24460.1, was downregulated while other mRNAs and targeted lncRNAs were upregulated in H-3n compared to M-3n and M-3nβ (Figure 5C). Given the results above, we constructed the core networks of lncRNAs–mRNAs that may function in *C. gigas* gametogenesis (Figure 5D–F, Table S8). The left and right hemispheres represent lncRNAs and mRNAs, respectively.

### 3.5. qRT-PCR Validation

The four lncRNAs chosen for validation were all randomly selected, and five genes, namely Cg-Vtg2 [28], Cg-Foxl2 [15], TESK2 [29], Pygb [30], and Tex36 [31] were reported in previous studies of *C. gigas* and mammals. The result confirmed that the qRT-PCR expression patterns were consistent with the RNA-seq data (Figure S1). It indicated that our pipeline was a robust method of identifying putative lncRNAs and that most of the identified lncRNAs were genuinely expressed in vivo.

## 4. Discussion

lncRNAs, functioning as a novel gene expression regulator, have received wide attention for their vital roles in mammal reproduction [3]. However, the regulatory mechanism of lncRNAs in marine invertebrates, with diverse reproductive patterns, remains elusive. In this study, we identified sterility-specific lncRNAs and constructed the core networks of lncRNAs–mRNAs. Combining with functional enrichment results, we explored the potential function of lncRNAs in the abnormal gametogenesis process. We hoped to provide a basis for future investigation regarding the mechanism of lncRNA regulation in the reproductive process of marine animals.

Gametogenesis is a delicate biological process accompanied by the regulation and expression of multiple related genes, whether in mammals or marine animals [32]. With plentiful fundamental research relating to the fecundity of triploids, *C. gigas* has been regarded as a potential model for molluscan species in studying gametogenesis [10,16]. Bioinformatic analyses from sterile and fertile oysters identified a large number of lncRNAs. Differential expression analysis indicated that these lncRNAs have distinct expression modules, underlining their potential roles in sterility. We identified 22 to 151 DELs and 675 to 1464 DEGs in the F-3nβ group, HF-3n group, and HM-3n group. Considering that triploid individuals of different genders may have diverse sterile patterns, we selected groups of DELs and DEGs for subsequent analysis.

The target genes of lncRNAs were predicted in the case of antisense and cis-regulatory mechanisms based on their physical positions and their coexpression relationships in the case of trans regulation. For the potential functions of DELs, the investigation was performed considering the three regulatory mechanisms mentioned above. Our study showed that antisense-target genes in the HF-3n group were enriched in progesterone-mediated oocyte maturation, cAMP signaling pathway, apoptosis, and TNF signaling pathway, which play a functional role in oocyte meiosis and maturation [5,33]. We found that TRPM3 is a cis-target gene of lncRNA MSTRG.19956.1 for both the F-3nβ group and the HF-3n group. It was demonstrated that TRPM3 was expressed in the spermatogenic cells and spermatozoa of rats and mouse testis, indicating its potential physiological function in germ cells [34]. Additionally, Tex36, cis-targeted by lncRNA MSTRG.22725.2 in the HF-3n group and the HM-3n group, was well elucidated in the gametogenesis process of mammals [31]. The low expression of TRPM3 and Tex36 may be associated with the small number of gametes in sterile individuals. Poor functional enrichment results of genes involved in cis and antisense regulation make it difficult to identify sterility-specific core genes and lncRNAs unambiguously.

To circumvent this problem, we shifted the focus towards trans regulation. In our work, groups of DEGs targeted by trans-action DELs showed identical genetic profiles based on Gene Ontology analysis for the top two subcategories. These DEGs were also mapped into KEGG pathways, and the top 20 statistically significant KEGG classifications are shown in Figure 4. Transcriptomic analysis of *C. gigas* unveiled that cell cycle and energy conservation difference was the primary cause of triploid sterility [17]. Given that, we focused on significantly enriched KEGG terms of interest. It was reported that cell cycle, oocyte meiosis, base excision repair, and progesterone-mediated oocyte maturation were essential for gonad development and gametogenesis of Tridacna squamosa and chicken [35,36]. Recent studies demonstrated that downregulation of the wnt signaling pathway was associated with delayed maturation of porcine oocytes and cell cycle arrest of mice granulosa cells [37]. In red swamp crayfish *Procambarus clarkia*, the p53 signaling pathway was independent for cell proliferation in late ovarian development [38]. The Jak-STAT signaling pathway was pivotal in regulating cell fate and necessary in germ cell proliferation of spotted knifejaw [39]. In Drosophila, the Hippo pathway affected cell proliferation in adult ovarian follicle cells, implying that gonadal somatic cells were functionally important for gametogenesis [40]. The KEGG pathway of the mRNA surveillance was closely related to gamete generation, as described in a single-cell RNA sequencing analysis of sheep spermatogenesis [41]. Energy supply is vital for the development of germ cells. Previous studies have demonstrated that the FoxO signaling pathway triggers diapause of insects likely through total carbohydrate decline induction [42]. By tracing the differentially expressed genes involved in the above signaling pathways, we can further understand the possible mechanism of trans-lncRNA in oyster gametogenesis.

Taking trans-analysis as the primary method, we established the core lncRNA–mRNA networks that may be associated with abnormal gametogenesis of triploid sterile females and hermaphrodites. In these core regulatory networks, sterility- or gametogenesis-specific genes were linked to their regulatory lncRNAs. We observed several cell-cycle-related factors in three groups, such as Cdc20, ccnb2, CCNA2, Cdc6, CYCE, CDK2, Bub1b, spdya-b, and their regulatory lncRNAs, MSTRG.79882.3, MSTRG.79882.4, MSTRG.63844.1, MSTRG.79882.2, MSTRG.70802.1, MSTRG.22725.2, MSTRG.33704.1, MSTRG.37949.1, MSTRG.50759.2, MSTRG.5675.2, MSTRG.68167.1, MSTRG.70320.1, MSTRG.5675.1, MSTRG.56971.1, and MSTRG.5736.1. As expected, those lncRNAs and their target genes were all downregulated in sterile individuals. Cell cycle regulators were essential for germline cell specification and gametogenesis [43]. The present study demonstrated that downregulated cell-cycle-related genes related to trans-lncRNAs above might contribute to gametogenesis arrest of triploids.

In the F-3nβ group, 12 DEGs, which participated in five pathways of statistical significance, were downregulated in F-3nβ, except BNIP3 targeted by MSTRG.9987.2. We identified that MSTRG.79882.4 and MSTRG.79882.3 interacted with most of the core genes, including KNDC1, dkf-2, AHNAK2, CELSR1, ft, ds, Axin1, NXF1, CCNA2, and CYME_CMM237C. Knockdown of KNDC1 was reported to promote cell proliferation [44]. This is in contrast to our data demonstrating low expression in sterile individuals. However, other potential mechanisms by which KNDC1 participates in reproduction cannot be excluded. Studies of CELSR1, ft, and dkf-2 have demonstrated their functions in inhibiting cell apoptosis, indicating their vital role in reproduction [45,46]. In addition, Axin1, which was required for the cell cycle process and proper spindle formation, was well characterized in mammal gametogenesis [47]. Cbp80 was predicted to interact with MSTRG.67996.1. NXF1 and Cbp80 are responsible for nuclear mRNA processing, export, stability, and quality control events and were verified to cause dominant sterility and germline-cell maintenance of Drosophila [48,49]. MSTRG.9987.2 and its target gene BNIP3, a critical signaling factor responsible for mediating programmed cell death, were upregulated in F-3nβ [50]. Interestingly, dkf-2, AHNAK2, CELSR1, ft, ds, Axin1, NXF1, CCNA2, and CYME_CMM237C were also identified when hermaphrodites were compared to fertile females, targeted by MSTRG.22725.2, MSTRG.33704.1, MSTRG.63844.1, MSTRG.70802.1, MSTRG.79882.3, and MSTRG.79882.4. This finding confirmed that the regulation of lncRNAs on sterility-related genes showed gender-specific differences and similarities. Among them, MSTRG.33704.1 and MSTRG.63844.1 were predicted to regulate 18 and 15 genes of 34 DEGs in the HF-3n group, respectively.

In the HM-3n group, MSTRG.21735.1 and MSTRG.28339.2 were predicted to regulate PYGM, which is involved in the insulin signaling pathway and was downregulated in hermaphrodites. Glycogen phosphorylase catalyzed glycogen decomposition likely reduced the deleterious effects of glycogen overproduction on germ cells of fertile individuals [51]. BOLL belongs to the DAZ gene family, which is required to establish germ cell identity, regulate germ cell development, and prepare germ cells for entry into meiosis [52]. Our RNA sequencing assay showed that the downregulation of BOLL and its regulatory lncRNA MSTRG.5675.1 might particularly impact the gametogenesis process of oysters. The process of meiosis is triggered by the formation of double-strand breaks, which must be repaired by homologous recombination subsequently. A previous study added to the evidence that structural maintenance of chromosome proteins was required for sister-chromatid-mediated recombination repair [53]. As a member of serine/threonine-protein kinase, plk1 contributes to DNA damage response and repair [54]. The possibility that sterility is due to errors in the germ cell meiosis program, thus, cannot be ruled out. In line with this conclusion, we found that smc3, smc1a, and plk1, regulated by MSTRG.55948.1, MSTRG.5675.1, MSTRG.40959.1, MSTRG.5736.1, MSTRG.79356.1, MSTRG.37949.1, MSTRG.5675.2, and MSTRG.70320.1, were downregulated. Herein, MSTRG.26314.7 and MSTRG.26316.1 were predicted to downregulate IQUB, which was reported uniquely present in sperm flagella [55]. This result echoed a previous report that flagella defects may be linked with spermatogenesis blocking [17]. MSTRG.5675.1 was predicted to interact with 6 genes of 17 unique DEGs in the HM-3n group, indicating it could play an important role. However, whether these transcripts are targeted by predicted lncRNAs or mediate their roles in the gametogenesis of *C. gigas* requires further investigation.

## 5. Conclusions

In summary, our results presented here demonstrated that sterility-related lncRNAs of *C. gigas* might mainly act via trans-regulatory functions. We identified 28 candidate trans-regulatory lncRNAs and 54 target genes that are involved in the pathways related to cell proliferation, apoptosis, and energy metabolism. These results revealed that trans-lncRNA-mediated regulation of sterility might be associated with cell-cycle arrest, germline-cell identity maintenance, and glycogen biosynthesis. Eventually, we constructed lncRNA–mRNA core networks in the F-3nβ group, the HF-3n group, and the HM-3n group. Although the involvement of gene expression in the regulation of lncRNA cannot be ruled out, the results of our study provide new insight for unearthing lncRNA-mediated mechanisms regulating the sterility of triploids.

## Figures and Tables

**Table 1:** Primer nucleotide sequences used in this study.

Transcript Name	Primer Sequence (5′ to 3′)	Amplicon Length (bp)	Amplification Efficiency
MSTRG.28339.2	F: TGTAGCAATGGGCAAACCAGA R: GCTAGGCCAGGTCCACTAAC	109	1.930
Tex36	F: TCCCGTAGATGCCGATTT R: AGGACTTGGGTCGGTGTTC	88	2.002
MSTRG.39041.2	F: TGTTTTGGACAAACCCAACGG R: TAATACGGTCACCGCAGCAT	131	2.186
MSTRG.22725.2	F: TGCTTCAAGACCAAATGCGG R: AGTCCCGCGGACATTTACAG	118	2.074
MSTRG.3451.1	F: TAAATGTGGAGGACCACGCC R: ACGACCATGACCTTTGCCTC	121	1.934

## Data Availability

The data presented in this study are available on request from the corresponding author.

## Supplementary Materials

The following supporting information can be downloaded at: https://www.mdpi.com/article/10.3390/biology11030378/s1, Figure S1. Validation of randomly selected genes and lncRNAs by qRT-PCR analysis; Table S1. The infor-mation of data quality control and sequence alignment for each sample; Table S2. The identifica-tion of differentially expressed lncRNAs in F-3nβ-vs-F-2n, F-3nβ-vs-F-3nα, H-3n-vs-F-2n, H-3n-vs-F-3nα, H-3n-vs-M-2n, H-3n-vs-M-3nα; Table S3. The identification of differentially ex-pressed genes in F-3nβ-vs-F-2n, F-3nβ-vs-F-3nα, H-3n-vs-F-2n, H-3n-vs-F-3nα, H-3n-vs-M-2n, H-3n-vs-M-3nα; Table S4. The prediction of the trans-regulatory relationship between lncRNAs and mRNAs; Table S5. The prediction of the cis-action relationship between lncRNAs and mRNAs; Table S6. The prediction of the antisense-regulatory relationship between lncRNAs and mRNAs.
